# Depressive Symptoms and Epistemological Approaches: A Qualitative Study Among Psychiatrists: Symptômes dépressifs et approches épistémologiques : une étude qualitative auprès de psychiatres

**DOI:** 10.1177/07067437251317245

**Published:** 2025-02-16

**Authors:** Clémence Piaux, Céline Bourquin, Friedrich Stiefel

**Affiliations:** 1 Service de psychiatrie de l’adulte nord-ouest, 30635 Centre Hospitalier Universitaire Vaudois et 27213 Université de Lausanne, Lausanne, Suisse; 2 Service de Psychiatrie de liaison, 30635 Centre Hospitalier Universitaire Vaudois et 27213 Université de Lausanne, Lausanne, Suisse

**Keywords:** qualitative study, psychiatry, depression, psychiatric nosography, diagnosis, treatment, epistemology, étude qualitative, psychiatrie, dépression, nosographie psychiatrique, diagnostic, traitement, épistémologie

## Abstract

**Background:**

The study aimed to investigate how psychiatrists handle the diagnosis and treatment of depression in the adult.

**Participants:**

Psychiatrists (*N* = 17) of the French-speaking Canton of Vaud (Switzerland), working in public institutions or in private sector (cabinets), having different theoretical backgrounds depending on their training (systemic, cognitive-behavioural or psychodynamic) and different duration of clinical experiences were included in the study.

**Methods:**

A clinical vignette presenting a young man with depressive symptoms of moderate intensity having experienced multiples losses during the development and recent past was presented to the participants. Participants were invited to read the vignette and react (“thinking aloud”), followed by an invitation to elaborate on the diagnosis and treatment.

**Results:**

A heterogeneous approach towards the diagnosis and treatment of depression was observed. Without a consensus regarding the diagnosis of depressions, two distinct ways to understand the clinical vignette emerged: one pathogenic, identifying the underlying causes of the depressive symptoms, and the other nosological, based on diagnostic criteria. Consequently, proposition for treatment also diverged ranging from psychotherapy, psychotropic medication, complementary and alternative treatments, and paramedical approaches such as ergo- and socio-therapy, at times leaving the choice up to the patient. Possible explanations for this diversity are a more or less strict adherence to diagnostic criteria used by the psychiatrists, be they ICD or DSM, the double training as psychiatrist and psychotherapist which is mandatory in Switzerland, a certain prudence regarding psychiatric diagnoses by fear of stigmatisation, attention to the therapeutic alliance or divergent views on the theoretical and conceptual understanding of depression.

**Conclusion:**

The results of this study underline the importance to include the epistemology of psychiatric disorders in training to raise awareness and conscientization regarding the influence of epistemological aspects on attitudes and approaches to the diagnosis and treatment of depression and other psychiatric disorders.

## Résumé

**Objectif:**

Cette étude qualitative vise à investiguer comment les psychiatres conçoivent le diagnostic et le traitement de la dépression.

**Participants:**

17 psychiatres exerçant dans le Canton de Vaud (Suisse), en public ou en privé, de différents horizons théoriques et avec expériences cliniques variables ont participé à l’étude.

**Méthode:**

Une vignette fictive présentant un tableau de dépression d’intensité moyenne était présentée aux participants. L’entretien commençait par la réaction du participant à la lecture de la vignette, suivie par deux questions concernant le diagnostic et le traitement. La méthode du *thinking aloud* a été utilisée pour le recueil des données qui ont été analysées au moyen d’une analyse thématique.

**Résultats:**

Il existe une hétérogénéité dans la manière dont les psychiatres approchent la dépression sur les plans diagnostique et thérapeutique. Une approche pathogénique et nosologique ont été identifié, avec des propositions de traitement très variables (psychothérapie, médication, traitements complémentaires et al.ternatifs ou paramédicaux), parfois laissés au choix du patient. Cette hétérogénéité peut s’expliquer par une adhérence plus ou moins stricte aux critères diagnostiques, la double formation en Suisse de psychiatre et de psychothérapeute, une certaine prudence quant au diagnostic en psychiatrie, l’attention portée à l’alliance thérapeutique ou les divergences de vue sur les origines de la dépression.

**Conclusion:**

Cette étude relève l’importance d’aborder la question de l’épistémologie et ses influences sur les attitudes diagnostiques et thérapeutiques dans la formation des futurs psychiatres.

« L’intérêt de la dépression est qu’elle se trouve dans une situation paradoxale: alors que l’épidémiologie psychiatrique estime depuis 1970 qu’elle est le trouble mental le plus répandu au monde, les psychiatres considèrent, hier comme aujourd’hui, qu’elle est impossible à définir».^1^

## Introduction

La dépression touche au niveau mondial plus de 300 millions de personnes, et représente la première cause d’incapacité de travail.^2^ Ce diagnostic est défini par certains critères décrits dans la classification internationale des maladies, CIM-10, et dans le Manuel Diagnostic et Statistique des troubles mentaux, DSM 5. La prise en charge fait l’objet de recommandations internationales^3,4^ et une abondante recherche scientifique s’y intéresse.

La question posée dans cette étude est celle de la représentation de la dépression chez les psychiatres. La littérature montre des différences de pratique entre psychiatres et médecins généralistes.^5–7^ Par exemple, les résultats d’une étude anglaise indiquent que les psychiatres n’utilisent pas les grilles diagnostiques, alors qu’ils les recommandent aux médecins généralistes.^5^ Selon une étude qualitative danoise, les généralistes se montrent sceptiques quant à l’utilisation d’échelles, alors que les psychiatres les utilisent pour évaluer la sévérité.^6^ Gigling et al. ont, ont montré que pour poser un diagnostic, les psychiatres se réfèrent peu à la sémiologie, contrairement aux généralistes^7^ et qu’ils accordent plus d’importance au soutien et aspects relationnels que les médecins généralistes.^7^

Des divergences sont aussi décrites parmi les psychiatres. Certains considèrent que les circonstances d’apparition des symptômes sont insuffisamment prises en compte et qu’un diagnostic de dépression ne devrait pas se limiter à la présence de symptômes et leur intensité,^8^ contrairement à ce qu’avancent les experts.^9^ D’autres remettent en question la validité des échelles,^10^ alors qu’elles sont recommandées.^3^ Enfin, un débat virulent a eu lieu lors de la parution du DSM 5 qui ne fait plus du deuil un critère d’exclusion de la dépression.^11^

En dehors de la communauté médicale, ce diagnostic est remis en question par certains sociologues^12^ qui estiment que les psychiatres ont tort de qualifier certains états affectifs de dépression. Ils constatent une médicalisation excessive de la tristesse,^13^ qui serait due à des changements sociétaux, comme l’épuisement lié à l’accélération sociale,^14^ la diminution du sentiment d’être émotionnellement relié au monde,^15^ le transfert de responsabilités professionnelles et existentielles sur l’individu,^16^ et les constructions sociales et politiques des émotions.^17^ Ils soulignent le rôle de l’industrie pharmaceutique dans la promotion des antidépresseurs et dans l’abaissement du seuil diagnostic.^18^

Cette étude s’intéresse à la manière dont les psychiatres-psychothérapeutes (i) approchent le diagnostic de dépression d’intensité moyenne et son traitement et (ii) à leur raisonnement. A notre connaissance, les représentations et le processus de diagnostic des psychiatres n’a fait l’objet que de deux recherches. La première, conduite auprès de huit internes débutant en psychiatrie en France, porte sur la formation d’une représentation de la dépression au cours de l’internat.^19^ Elle montre qu’une partie de la formation des internes consiste à se détacher de l’idée d’une entité fixe et à ne pas retenir des états psychiques comme dépression, alors qu’ils l’auraient fait avant leur spécialisation. L’objectif de la seconde recherche, réalisée en Suisse,^20^ était d’analyser les représentations de la dépression chronique qu’ont les psychiatres, le traitement qu’ils proposent, et leur vécu face à ce trouble. Elle montre que leurs représentations se basent sur les classifications internationales, mais qu’ils les jugent insuffisantes. Ils se référeraient davantage au « fonctionnement psychique » et aux aspects sociaux pour comprendre ces situations et proposer une prise en charge.

## Méthode

### Setting et participants

L’étude a été conduite dans le Canton de Vaud, en Suisse. Les participants sont des psychiatres-psychothérapeutes installés, identifiés dans l’annuaire de la Société Vaudoise de Médecine, ou médecins cadres dans les services de psychiatrie publique. Il s’agit de psychiatres expérimentés qui ont en moyenne quinze années d’expérience après le diplôme de spécialiste, ce dernier nécessitant au moins six années de formation post-graduée. Nous avons fait ce choix afin d’avoir des participants dont on peut supposer qu’ils ont un recul par rapport à l’enseignement reçu et une habitude à pratiquer. Leur sélection, aléatoire, visait à représenter l’ensemble des psychiatres de cette région, en termes notamment de genre, âge, lieu de formation et de pratique, et d'orientation psychothérapeutique. Nous avons exclu les pédopsychiatres et psychiatres de l’âge avancé, en raison de la spécificité des tableaux cliniques de dépression à ces âges. Parmi les 18 psychiatres-psychothérapeutes contactés, un a refusé de participer à l’étude. Les participants sont âgés de 35 à 70 ans (médian 52 ans), 6 sont des femmes, 14 pratiquent en ville et 3 à la campagne, 8 en cabinet privé, 6 rattachés au service de psychiatrie publique et 3 ont une activité mixte - cabinet privé et psychiatrie publique. 11 sont d’orientation psychodynamique, 4 systémique et 2 cognitivo-comportementale. Tous ont effectué leur formation post-graduée en Suisse. En ce qui concerne le contexte, le Canton de Vaud compte environ 800’000 habitants et la psychiatrie publique y est organisée selon le modèle de « secteur », avec quatre secteurs géographiques distincts. Celui dit du « centre » comprend la ville de Lausanne et sa périphérie, les trois autres les régions plus rurales (Nord, Ouest et Est). Les dispositifs hospitaliers et ambulatoires publiques de trois secteurs sur quatre, dont le secteur centre qui est le plus académique, font partie du Département de psychiatrie qui est intégré dans le Centre Hospitalier Universitaire Vaudois (CHUV). Une fondation privée d’intérêt publique gère le secteur Est. La psychiatrie privée est assumée par des psychiatres installés en cabinet. Nous avons interrogé des participants exerçant dans les quatre secteurs, en psychiatrie publique et privée, à l’hôpital et en ambulatoire. La Suisse compte par ailleurs l’une des densités de psychiatres les plus élevées au monde (51 pour 100’000 habitants), en comparaison avec l’Allemagne (24 pour 100’000 habitants) ou le Canada (13 pour 100’000 habitants).^21,22^

### Production des données

Des entretiens semi-directifs ont été conduits sur leur lieu de travail ou via une visioconférence.

Le guide d’entretien a été élaboré autour d’une vignette clinique, conçue comme support à la réflexion. Elle a été tirée d’un article sur le suicide^23^ (voir tableau 1) et traduite en français. Dans l’étude originale, la vignette servait à comparer l’issue d’une consultation d’un patient déprimé avec celle d’un patient suicidaire. La description clinique se base sur l’échelle de Beck.^24^ Quelques modifications ont été apportées pour adapter la vignette au contexte local, ainsi que des ajouts (phrase soulignée ci-après) afin que la vignette remplisse les critères CIM-10.

**Tableau 1.** vignette clinique adaptée (Leysin est un village de montagne, Lausanne est la ville principale du canton de Vaud, un CFC est le diplôme d’une formation professionnelle en Suisse).

Pierre est un homme de 31 ans, célibataire sans enfant, qui vit seul. Il vient de commencer une psychothérapie. Son histoire est la suivante : Pierre est un enfant unique, sa mère est décédée d’un cancer quand il avait 10 ans. Il a vécu avec son père à Leysin jusqu’à ce qu’il termine son CFC (certificat fédéral de capacité de travail), puis il a dû déménager à Lausanne pour trouver un emploi. Au bout de cinq ans, son patron le licencie. Il y a un mois, après une période de disputes incessantes, sa petite-amie lui annonce qu’elle ne l’aime plus et ils se séparent. Au cours du dernier mois Pierre s’est senti triste, désespéré à propos de son avenir et a de la difficulté à envisager le futur. Il pleure souvent, a une certaine perte de plaisir, se sent sans valeur et se vit comme un échec, ce qui aggrave sa déception. Il ne dort pas bien la nuit, se réveille souvent et rencontre des difficultés à se rendormir.

Selon la CIM-10 avec 2 critères typiques et 4 atypiques, la vignette décrit un cas de dépression d’intensité moyenne, contrairement au DSM-5 (seuls 4/5 critères nécessaires sont présents). Cette discordance a été volontairement maintenue car pouvant constituer un sujet de discussion. Enfin, nous avons choisi de ne pas utiliser un cas de dépression sévère, faisant l’hypothèse que les questions de diagnostic et de traitement seraient moins sujettes à discussion.

Le guide d’entretien s’articule autour de deux parties. La première est une question ouverte visant à recueillir la réaction spontanée du participant à la lecture de la vignette, ce que nous avons appelé la « porte d’entrée du raisonnement clinique ». Dans la deuxième partie deux questions plus spécifiques portent sur le diagnostic et le traitement.

Pour les deux parties nous avons demandé aux participants de penser à haute voix (*thinking aloud method*),^25^ c’est-à-dire de rapporter spontanément lors de la lecture de la vignette puis lorsque les questions leur sont posées, en leur demandant de ne pas chercher à interpréter ou à analyser leurs réflexions. D’une durée de 20 à 30 min, les entretiens ont été conduits par la première auteure (CP), audio-enregistrés, et intégralement transcrits.

### Analyse des données

Les données ont été analysées au moyen d’une analyse thématique visant à repérer et regrouper les dimensions principales de la réflexion et du raisonnement des participants.^26^ L’analyse s’est focalisée sur les thèmes saillants rapportés à la suite de la lecture de la vignette, pour ensuite porter spécifiquement sur les questions diagnostiques et thérapeutiques. Les analyses ont été conduite par la première auteure (CP) et revues et discutées en équipe avec les deux co-auteurs. CP et FS sont des psychiatres cliniciens, tandis que CB est une chercheuse issue des sciences sociales intégrée depuis longtemps au sein d’un Service de psychiatrie de liaison.

### Éthique

La Commission cantonale d'éthique de la recherche sur l'être humain du Canton de Vaud (CER-VD) a considéré que l’étude n’entre pas dans le champ d’application de la LRH (Loi relative à la recherche sur l’être humain) car elle ne récolte pas de données personnelles liées à la santé selon l'art.2 LRH. L’étude a donc été exemptée d’approbation, mais elle respecte néanmoins les droits des participants, en termes d’information, de consentement et de respect de la confidentialité (éthique de la recherche). Tous les participants ont ainsi été informés des buts de l’étude et ont donné leur consentement écrit et oral.

## Résultats

Nous allons présenter les « portes d’entrée du raisonnement clinique » puis détailler les résultats liés aux diagnostic et traitements. Des extraits d’entretiens sont utilisés à des fins d’illustration.

### Thèmes portes d’entrée du raisonnement clinique

Les premiers contenus amenés par les participants peuvent être regroupés en quatre thèmes : perte, crise, diagnostic et symptômes. La perte et la crise sont deux concepts pathogéniques alors que les « diagnostic » et « symptômes » se situent du côté d’un raisonnement médical.

La perte est évoquée sous l’angle de son accumulation, « des pertes comme ça multiples » (P6), ou de pertes passées, durant le développement, produisant un effet d’écho, « un contexte lié à des changements et des séparations et qui peuvent réactiver la perte qu’il a connue à ses 10 ans » (P14). Le thème crise prend valeur diagnostique chez certains participants, « je perçois qu’actuellement il est en crise » (P17), ou une question à explorer chez d’autres, « quelle est la crise actuelle» (P15).

Ceux qui commencent par le diagnostic ou des symptômes évoquent d’emblée le registre dépressif, « un premier épisode dépressif » (P1), « toute la symptomatologie dépressive » (P4).

### Diagnostic

Environ la moitié des participants retiennent un diagnostic de dépression, utilisant les classifications CIM et DSM, « un tableau de dépression majeure » (P16), les classifications plus anciennes, « une dépression réactionnelle » (P17) ou le terme générique de « dépression » (P3).

D’autres hésitent, concernant la présence de symptômes dépressifs, « il faudrait que j’investigue, mais à vue de nez, on peut imaginer qu’il y a des symptômes dépressifs » (P11), ou leur nombre, « il manque des éléments psychiatriques comme ça pour déterminer un épisode dépressif majeur ou pas » (P12). La question du diagnostic différentiel est aussi abordée, « oui le tableau dépressif certainement. Après, le diagnostic final, est-ce que c’est un trouble dépressif récurrent, un épisode inaugural, est-ce qu’il y a des comorbidités…» (P15).

#### Raisonnement autour du diagnostic

Les participants se différencient s’agissant de l’importance donnée au diagnostic. Pour certains, il est très important en soi, « j’ai besoin de clarifier un certain nombre de points (…), d’un moment d’entretien semi-structuré (…) d’une formulation de cas » (P15), car « ma valence médicale est bien là » (P15). Pour d’autres il est important, mais investi avec détachement, « tout simplement je vais compter les symptômes CIM ou DSM, tac, tac, tac […] » (P1), ou pour justifier certains actes, « s’il faut prescrire un traitement et puis écrire un rapport pour une assurance, définitivement je retiendrais un épisode dépressif » (P8). Pour les derniers, la question ne se pose pas vraiment, « ce n’est pas un enjeu dans ma pratique de vouloir savoir rapidement si c’est un épisode dépressif » (P5). Finalement, il y a aussi une utilisation différente des critères diagnostiques, un participant s’intéressant à leur persistance pour pouvoir retenir le diagnostic, et un autre au contexte culturel : « si ce jeune s’était présenté à un psychiatre en Grèce, le psychiatre lui aurait dit pars, tu n’as rien » (P8).

L’impact du diagnostic sur la relation thérapeutique est pris en compte avec le souhait de se mettre d’accord sur le diagnostic, et avec la crainte de faire du patient un « malade », « il y a quand même le fait que […] juste après un licenciement et après une séparation… il y a une tristesse normale » (P6).

### Traitement

La totalité des participants proposent une psychothérapie, avec environ un quart associé d’un traitement médicamenteux, paramédical (ergothérapie, sociothérapie) ou issu des médecines complémentaires.

La psychothérapie proposée varie selon l’axe d’orientation du thérapeute, mais l’objectif rapporté est proche, « mon premier traitement serait d’essayer de trouver du sens » (P17, axe psychodynamique), « donner sens à ce qui lui arrive et voir comment, qu’est-ce qu’il peut mettre en place lui aussi pour faire en sorte d’aller mieux » (P9, axe systémique), « pouvoir donner un sens à ce qui arrive » (P4, axe TCC). Ensuite, certains, dans les trois axes, évoquent une psychothérapie à long terme visant un changement durable du fonctionnement psychique. Il existe une différence entre ceux qui proposent une psychothérapie après les premiers entretiens d’évaluation, et ceux qui considèrent que les premiers entretiens sont déjà psychothérapeutiques.

Quelques participants se positionnent d’emblée pour ou contre un traitement antidépresseur, « je serais assez rapidement pour le traiter » (P3) ou « d’abord une IPB (intervention psychodynamique brève) de toute façon sans médicament » (P10). La majorité cependant ne se prononce pas. Certains estiment le traitement pharmacologique indiqué pour des formes de dépression sévère (cette éventualité ne peut être ni confirmée ni exclue en lisant la vignette), d’autres le prescriraient à la demande du patient ou si les premiers entretiens n’apportent pas une amélioration.

Un participant propose d’autres approches, ayant comme but de sortir le patient d’un supposé isolement, telles que l’ergothérapie et la sociothérapie. Certains proposent des thérapies issues des médecines alternatives et des mesures d’hygiène de vie, « des mesures pour le régime de vie, des méthodes de relaxation, du floating, de la sophrologie, de l’acupuncture… » (P3).

Enfin l’un dernier participant réfléchit à une hospitalisation, tout en la réfutant, « Est-ce qu’il y aurait une indication à ce qu’il soit protégé, une hospitalisation ? Ça ne me parait pas le plus probable mais quand même » (P16).

#### Raisonnement autour du traitement

La psychothérapie est proposée car « suffisante », « il a débuté sa psychothérapie donc je lui proposerais de la continuer déjà. Et puis souvent ça suffirait » (P6), ou parce qu’elle permet de réfléchir aux relations interpersonnelles, « je priorise la psychothérapie (…) parce que c’est celle qui permet la prise en charge de la dimension relationnelle, dans tout ce qui se passe pour lui celle-ci est assez évidente » (P7), et une maturation ultérieure, « une psychothérapie ça peut l’aider aussi à découvrir autre chose plutôt que de l’enfermer » (P8). Plusieurs proposent une approche initiale structurée, cependant la suite du traitement reste ouverte, « on a toujours des suivis de thérapie qui se rallongent » (P3), ou, « on peut espérer qu’après quatre entretiens c’est réglé (P10) ».

Les participants soulignent la nécessité de créer un lien, « les objectifs que j’ai c’est établir une alliance thérapeutique (…) ça c’est la base » (P4), et de s’entendre avec le patient sur le traitement. Enfin on retrouve la crainte de médicaliser une tristesse « normale » : « c’est légitime de réagir, tout n’est pas un diagnostic de dépression et tout ne nécessite pas forcément de mettre d’emblée des molécules » (P14).

Enfin certains aspects sanitaires sont considérés, par exemple l’offre de soins locale, « on a de la chance en Suisse de pouvoir proposer ce côté psychothérapeutique etc., dans d’autres pays ce n’est pas possible. Le psychiatre va le voir 10 min, prescrire un médicament et c’est bon » (P1).

## Discussion

Les résultats montrent une hétérogénéité dans la façon dont les psychiatres approchent le diagnostic et le traitement de la dépression.

Nous commençons par contextualiser notre étude puis discuterons les résultats. En effet, la Suisse présente la particularité depuis 1961 d’une double formation et titre en psychiatrie et en psychothérapie. Cela signifie qu’au cours des cinq années de spécialisation en psychiatrie, les médecins décident dans quel axe (psychanalytique, cognitivo-comportemental ou systémique) ils souhaitent se former. Pour obtenir le titre de spécialiste, ils doivent effectuer 300h de séances de psychothérapie, 150h de supervision à la psychothérapie, 80h de psychothérapie personnelle, et suivre des séminaires théoriques au sujet de la psychothérapie. Beaucoup de psychiatres-psychothérapeutes dépassent par ailleurs largement les 80 heures de psychothérapie personnelle et continuent d’être supervisés après l’obtention du titre. Ainsi, le traitement psychothérapeutique est souvent pratiqué en première intention.^27^ En Suisse, il est donc courant de se rendre chez un psychiatre pour entamer un travail psychothérapeutique. Enfin, les assurances remboursent la psychothérapie au long cours (10% des frais sont à la charge du patient), si un diagnostic est posé.

La vignette fut abordée de deux manières distinctes, la première pathogénique et la seconde nosologique. Cette différence ramène à un débat historique^28^ avec d’une part Freud, qui cherche une cause psychologique aux troubles mentaux liée à l’inconscient, dans un continuum avec le fonctionnement normal; et d’autre part par Kraepelin qui s’oriente aux symptômes manifestes et à la classification qui distinguent le pathologique du normal. Cette polarisation se retrouve dans nos résultats.

Concernant le diagnostic, nous pouvons relever que l’évolution du DSM est allée vers une réduction des critères, comme l’a montré Crocq.^29^ Une hypothèse pourrait être qu’actuellement cette simplification est allée trop loin, nécessitant pour les psychiatres d’inclure d’autres paramètres, par exemple le contexte socio-culturel, à leur raisonnement, ce que Linder et al. ont déjà montré pour la dépression chronique.^20^ Cette évolution se fait rapidement (environ 60 ans), ce qui pourrait expliquer que les psychiatres portent différemment en eux des « traces » de ces époques, comme en témoignent une référence à la dépression « réactionnelle » (valable jusqu’au DSM-III, 1978). Enfin les critères peuvent être discordants entre les deux manuels, générant des différences selon l’utilisation de l’un ou l’autre, tel que dans la vignette présentée. Les divergences de conclusion entre les deux systèmes de classification peuvent donc générer des attitudes différentes chez les psychiatres. De surcroît, l’utilisation des critères des classifications et/ou des recommandations pour le traitement ne faisaient pas consensus chez les participants.

La crainte de stigmatiser le patient est aussi une préoccupation évoquée. Link et Phelan^30^ identifient quatre étapes dans ce processus, dont la première est d’étiqueter qui signifie identifier et nommer une différence. Certains psychiatres semblent penser que poser un diagnostic de dépression équivaut à « étiqueter », d’où leur réticence. A l’inverse cette crainte semble inexistante lorsque le diagnostic permet faire recours aux assureurs et justifier un traitement.

Ce qui est frappant c’est que les participants semblent défendre leurs propos de manière « binaire » comme si un diagnostic avait soit une importance majeure, soit aucune importance.

En ce qui concerne le traitement, il y a un consensus sur la proposition d’une psychothérapie, souvent associée à une référence à l’alliance thérapeutique. Pour certains les premiers entretiens sont thérapeutiques, alors que d’autres les considèrent comme une évaluation, de nouveau, comme si l’un excluait l’autre. Les points de vue divergent s’agissant de la médication et des approches paramédicales et complémentaires, avec certains proposant une multitude d’options parmi lesquelles le patient devrait faire son choix. Ces différences pourraient être liées au lieu d’exercice (avec des offres thérapeutiques variables), à la diversité des parcours de formation et aux orientations psychothérapeutiques. De plus, les représentations au sujet d’un traitement médicamenteux peuvent varier, comme nous l’avions discuté dans un article de réflexion. Nous y relevions notamment les motivations psychologiques menant le thérapeute à favoriser ou rejeter les traitements médicamenteux.^31^ Dans la présente étude, nous constatons que les orientations théoriques influencent également la proposition d’une prescription de psychotropes. Par exemple, l’intervention psychodynamique brève écarte toute prescription lors des quatre séances d’investigation initiale et le constat d’une dépression moyenne selon la CIM conduit certains participants à suivre les recommandations et à prescrire un traitement dès le premier entretien. Alors que le fait que le patient reçoive le traitement qu’il désire est fortement corrélé au développement d’une alliance thérapeutique,^32^ ce que chaque psychiatre propose semble ici circonscrit par sa propre formation. Même si le propos est de laisser un libre choix au patient concernant le traitement, celui-ci prend des formes très différentes puisque l’offre proposée est très variable. Il est compréhensible que traiter d’emblée le patient avec un antidépresseur fasse débat au vu les doutes sur leur efficacité pour les états dépressifs d’intensité faible et moyenne et les effets placebo et nocebo des médicaments^33^ et notamment des antidépresseurs.^34^ Les médecines complémentaires ou paramédicales ne font pas partie des recommandations scientifiques, mais sont abondamment conseillées par les médias grand public (^35–37^). Enfin, la population générale ne se montre pas favorable aux traitements médicamenteux.^38^ Les psychiatres doivent alors considérer des positions contradictoires entre ce qu’ils retirent de leur formation (approche scientifique, théories psychothérapeutiques), leurs propres croyances et expériences, et les attentes des patients.

Un parallèle peut être fait entre l’étendue des traitements proposés et l’extension de la psychiatrie à la « santé mentale » décrite par Ehrenberg^1^ et Rechtman.^39^ Ehrenberg lie des états ressemblant à la dépression à l’évolution sociale avec la montée de la liberté (et responsabilité) individuelle,^16^ dont le corollaire est, pour lui, que la société doit offrir aux individus des soins couvrant la santé mentale au sens large. Quant à Rechtman, il constate que les questions relevant de la « souffrance psychique » sont désormais adressées aux psychiatres, alors que cette notion floue décrit paradoxalement « une accumulation de petits signes infracliniques ne permettant justement pas de poser un diagnostic psychiatrique ».^39^ Il montre qu’il ne s’agit pas de « psychiatriser » les personnes, mais de pouvoir proposer à cette population dite « normale », souffrant des changements sociaux, les soins de professionnels de la santé mentale.^39^

Enfin, notre étude comporte des limites. Premièrement, les entretiens ont été menées par l’investigatrice alors qu’elle était en formation dans la région où a été menée l’étude. Elle a suivi les enseignements de certains participants et en a côtoyé d’autres comme collègues, de manière directe ou indirecte. Cela a pu influencer la production des données. Certains participants ont en effet semblé réticents. D’autres ont adopté une attitude défensive, justifiant toutes leurs réponses de façon généraliste (théories psychanalytiques ou « guidelines »), ce qui ne correspond peut-être pas à la diversité de leurs pratiques. En outre, il serait intéressant d’identifier plus précisément les facteurs expliquant le raisonnement et les différentes approches proposées par les cliniciens-participants (contexte d’exercice, caractéristiques sociodémographique, effet de la formation, rapport à la psychothérapie ou aux médicaments, etc.). Cette question dépasse néanmoins le cadre de notre étude.

## Conclusion

Notre étude montre la grande diversité des manières d’aborder la dépression des psychiatres-psychothérapeutes, tant sur le plan diagnostic que thérapeutique. Est-ce que la dépression est en soi un objet flou, voire un objet frontière^40^ qui justifie des conceptions différentes selon le point de vue de chacun ? Ou est-ce que chaque regard et approche demeurent incomplets ? Est-ce que les divergences de représentations et propositions des psychiatres interrogés sont liées au vieux problème de l’esprit (*mind*) et du cerveau (*brain*)? Ou est-ce qu’elles sont explicables par le développement humain, au cours duquel les dimensions sociales viennent construire et nourrir nos états émotionnels? Il semble en tout cas que nos résultats montrent l’importance de faire figurer l’épistémologie médicale et psychiatrique dans la formation des futurs psychiatres.
